# Development of the attribution scale for behavioral problems in children with special educational needs: a reliability and validity study

**DOI:** 10.3389/fpsyg.2025.1679823

**Published:** 2026-01-13

**Authors:** Da Deng, Yanling Li

**Affiliations:** 1 School of Teacher Education, Chengdu University, Chengdu, China; 2 School of Education, Geely University of China, Chengdu, China

**Keywords:** attribution of behavioral problems, behavioral problems in children, children with special needs, reliability, validity

## Abstract

**Introduction:**

With the comprehensive advancement of inclusive education in China, the proportion of children with special educational needs (CSEN) enrolled in mainstream schools has continued to increase. The identification and intervention of behavioral problems among these students have become core challenges in the implementation of inclusive education. This study aims to develop the Attribution Scale for Behavioral Problems in Children with Special Educational Needs, tailored to the Chinese cultural context, and to examine its reliability and validity.

**Methods:**

A multi-stage sampling approach was adopted, involving teachers from both mainstream and special schools in Sichuan Province. First, in-depth interviews were conducted with 30 teachers (15 from mainstream schools and 15 from special schools) to generate the initial item pool. Second, a pilot test was administered to 32 teachers for item discrimination analysis and preliminary assessment of internal consistency reliability. Third, a formal questionnaire survey was conducted among teachers from 14 schools, yielding 195 valid responses (10 special education teachers, 185 mainstream teachers) for exploratory factor analysis (EFA). Fourth, a larger-scale survey was distributed to teachers from 31 schools, with 328 valid responses collected (35 special education teachers, 293 mainstream teachers) for confirmatory factor analysis (CFA) and further reliability testing. A comprehensive analytic strategy was employed, including critical ratio (CR) analysis, item-total correlation, EFA, CFA, and calculation of Cronbach’s alpha coefficients.

**Results:**

The finalized Attribution Scale for Behavioral Problems in Children with Special Educational Needs consists of 36 items. EFA identified five distinct factors: neurophysiological drivers, environmental-task adaptation, motivation-rule cognition, emotional–social interaction, and support system deficiencies. The Cronbach’s alpha coefficients for the total scale and each subscale ranged from 0.887 to 0.908, indicating high internal consistency. Correlations between the subscales and the total score ranged from 0.705 to 0.786 (*p* < 0.001). CFA demonstrated excellent model fit (CFI = 0.996, RMSEA = 0.012), with all factor loadings reaching statistical significance.

**Conclusion:**

The newly developed Attribution Scale for Behavioral Problems in Children with Special Educational Needs demonstrates a clear structure and robust reliability and validity. It offers a scientifically sound tool for the assessment and intervention of behavioral problems in CSEN within the context of inclusive education in China.

## 1 Introduction

In recent years, China’s special education sector has seen significant progress, driven by the advancement of inclusive education policies such as the “14th Five-Year Special Education Promotion and Enhancement Action Plan” (2021) and the deepening of inclusive education philosophy (Han and Deng, 2025). The enrollment of children with special educational needs (CSEN) in mainstream schools has surged, highlighting the growing diversity in their educational and behavioral support needs. Recent statistics indicate that over 700,000 CSEN are enrolled in compulsory education, with approximately 75% attending mainstream schools. Despite these advancements, challenges remain in identifying and intervening in behavioral issues—such as emotional disorders, aggressive behaviors, and attention deficits—which impede individual development and complicate classroom management. These issues represent a critical barrier to the high-quality development of inclusive education (Han and Lei, 2025; Zhu et al., 2018). The implementation of revised policies, including the “Regulations on the Education of Persons with Disabilities” (2017), has facilitated a shift in China’s special education from focusing solely on “ensuring access” to “improving quality.” (The The State Council of the People’s Republic of China, 2017).

Contemporary empirical research on behavioral interventions for CSEN, both domestically and internationally, predominantly centers on two main approaches: behavior modification techniques grounded in Applied Behavior Analysis (ABA; Cooper et al., 2020), and environment-based strategies such as Positive Behavior Support (PBS; Sugai and Simonsen, 2012). Comprehensive support schemes, such as the U.S. Individualized Education Program (IEP), integrate academic, social, and behavioral goals (Kurth and Mastergeorge, 2010), demonstrating significant improvements in academic and social outcomes for students with special needs (Yell et al., 1998). In the United Kingdom and Australia, theories such as ecological systems theory (Bronfenbrenner, 1979) and social cognitive theory (Bandura and Walters, 1977) have been employed to examine the mechanisms underlying behavioral issues among CSEN (Kirana et al., 2025). Notably, Kim et al. (2017) found that aberrant synaptic pruning in children with autism spectrum disorder (ASD) significantly affects behavioral adaptation, offering insights into the development of more precise intervention strategies. These studies collectively underscore that behavioral issues among CSEN arise from the interplay of biological, psychological, and social factors.

Accurate measurement tools are essential for identifying and intervening in behavioral problems among CSEN. While international instruments like the Child Behavior Checklist (CBCL) and Teacher’s Report Form (TRF) are reliable, their applicability within the Chinese context remains limited (Achenbach et al., 2008). Previous studies have highlighted the need for recalibrating these tools to optimize their sensitivity and specificity for Chinese populations (Su et al., 1997). Furthermore, these tools fail to adequately address culturally relevant dimensions, such as home–school collaboration and policy support (Yang et al., 2020). Despite the expansion of educational opportunities for CSEN through policies like “learning in regular classrooms” and resource room models, behavioral management remains a significant challenge (Singh et al., 2009). For example, Zhu et al. (2018) reported that 62.3% of students with special needs in inclusive classrooms experience behavioral problems, posing a substantial challenge to both teachers and students. Although efforts have been made to implement functional behavior assessment (FBA) and positive behavior support (PBS) in China, issues persist regarding the standardization, scope, and localization of assessment tools (Paclawskyj et al., 2001). Wang and Zheng (2024) noted a lack of unified standards in FBA and PBS, resulting in inconsistent evaluation outcomes. Additionally, Matson et al. (2012) found that existing interventions focus predominantly on severe behavioral problems, neglecting the broader spectrum of behaviors. Wang et al. (2015) further pointed out that the direct application of foreign tools faces significant challenges related to language, culture, and educational context, emphasizing the need for localized adaptations (Singh et al., 2006).

Theoretical explanations for behavioral problems among CSEN currently encompass five main perspectives: (1) ecological systems theory, which emphasizes the dynamic interaction between individuals and their family, school, and broader societal systems (Bronfenbrenner, 1979); (2) behaviorist models, which focus on the relationship between environmental stimuli and behavioral responses, utilizing techniques like positive reinforcement (Guo, 2019); (3) social support theory, which underscores the importance of resource integration and support systems in behavioral intervention (del Castillo, 2024); (4) cognitive developmental theory, which links cognitive biases to behavioral problems, advocating for cognitive training to enhance behavioral regulation (Yang et al., 2020); and (5) humanistic theory, which advocates for individualized education to meet the diverse developmental needs of CSEN and supports peer and teacher relationships (Guo, 2019).

Given these theoretical frameworks and the challenges identified in the field, there is an urgent need to develop culturally appropriate assessment tools for behavioral problems among CSEN in China. This study aims to address this gap by systematically developing the Attribution Scale for Behavioral Problems among Children with Special Educational Needs. Drawing from established theoretical frameworks and integrating data from open-ended questionnaires and in-depth interviews, this scale provides a rigorous and culturally relevant tool for assessing and intervening in behavioral problems among CSEN in China, advancing both empirical research and theoretical understanding in the field.

## 2 Objects and methods

### 2.1 Participants

#### 2.1.1 Sample 1

To conduct qualitative analysis, a total of 30 teachers were randomly selected from Sichuan Province, including 15 teachers from regular primary schools and 15 teachers from special education schools in Chengdu. These participants took part in in-depth interviews.

#### 2.1.2 Sample 2

For the pilot testing of the questionnaire, 32 teachers were randomly selected from Sichuan Province, including 20 from regular primary schools (62%) and 12 from special education schools (38%). Among them, 17 were class teachers (53%), and 15 were not (47%). Critical ratio (CR) values were calculated to assess item discrimination, item-total correlations were analyzed, and Cronbach’s alpha was used to examine the internal consistency reliability of the refined scale.

#### 2.1.3 Sample 3

For exploratory factor analysis (EFA), a questionnaire survey was distributed via the Wenjuanxing platform to teachers at 14 regular primary and special education schools in Sichuan Province. A total of 200 questionnaires were collected. After excluding invalid responses (e.g., patterned responses, completion time too short, or inconsistent answers to repeated items), 195 valid questionnaires were retained, resulting in an effective response rate of 97.5%. Among the respondents, 10 were special education teachers (5%) and 185 were regular primary school teachers (95%); 143 served as class teachers, accounting for 73.33% of the sample.

#### 2.1.4 Sample 4

This sample was used for confirmatory factor analysis and reliability and validity testing. Questionnaires were distributed to 31 primary and special education schools in Sichuan Province through the Wenjuanxing platform. A total of 338 questionnaires were collected. After excluding invalid questionnaires with regular responses, short response times, and score differences of more than 2 points for repeated questions, 328 valid questionnaires were received, with an effective rate of 97%. Among them, there were 35 teachers (11%) from special education schools and 293 teachers (89%) from regular primary schools. Of these, 248 served as class advisors, accounting for 75.6%.

### 2.2 Questionnaire compilation

#### 2.2.1 Qualitative analysis of the attribution of behavioral problems in children with special educational needs

Guided by ecological systems theory (Bronfenbrenner, 1979) and social cognitive theory (Bandura and Walters, 1977), which emphasize the complexity of person–environment interactions and the multifactorial mechanisms underlying behavioral problems, this study conducted in-depth interviews with Sample 1. The qualitative phase was further informed by domestic research approaches on functional behavior assessment (FBA) and positive behavior support (PBS).

Interview and open-ended questionnaire items focused primarily on teachers’ instructional contexts and classroom management practices, including:

1. Grade level, subjects taught, years of experience as a homeroom teacher, class size, and number of students requiring special attention;
2. Common disruptive behaviors observed in class and analysis of their underlying causes (e.g., student characteristics, fear of failure, task design);
3. Problems encountered by students in completing assignments across subjects and the contributing factors, including ability, interests, and task formats;
4. Frequent challenges in classroom management and analysis of the reasons behind students’ rule violations, such as deliberate defiance, confrontational behaviors, and imitation among peers;
5. Common conflicts observed during recess or lunch breaks and their antecedents, including students’ conflict-resolution skills, environmental factors, and leadership roles within peer groups;
6. Behavior modification strategies currently employed by teachers and their perceived effectiveness;
7. Types of support provided to teachers by the school;
8. Teachers’ expectations for support and the rationale behind these expectations.

With participant consent, interviews were audio-recorded. The interview transcripts and responses to open-ended questions were combined, transcribed, and coded using NVivo software. Initial screening of the qualitative data was followed by the categorization of responses into independent thematic units, which were then refined into core attributions. These core attributions were subsequently grouped based on thematic similarity and divergence, resulting in five distinct domains of behavioral attributions:

1. Neurophysiological Drivers,
2. Environmental and Task Adaptation,
3. Motivation and Rule Cognition,
4. Emotional and Social Interaction,
5. Multi-support Systems.

A summary of these five domains is presented in Table 1.

**Table 1:** Core attribution characteristics of children with special needs: qualitative analysis results.

Domain	Subcategory
1. Neurophysiological drivers	(1) Physical & neurodevelopmental disorders
	(2) Physiological needs & impulsivity
	(3) Cognitive & perceptual impairments
2. Environmental and task adaptation	(1) Task design & difficulty
	(2) Physical environmental constraints
	(3) Insufficient teaching resources
3. Motivation and rule cognition	(1) Motivation & attitude
	(2) Rule awareness & behavioral norms
4. Emotional and social interaction	(1) Emotional regulation& expression
	(2) Social skills & relationships
5. Multi-support systems	(1) Family support
	(2) School resources
	(3) Social support

#### 2.2.2 Item development for the attribution scale of behavioral problems in children with special educational needs

Based on a synthesis of prior literature and the findings derived from qualitative analysis, an initial item pool was developed for the Attribution Scale of Behavioral Problems in Children with Special Educational Needs (CSEN). First, item construction was informed by five theoretically grounded domains identified through qualitative coding: Neurophysiological Drivers, Environmental and Task Adaptation, Motivation and Rule Cognition, Emotional and Social Interaction, and Support System Deficits.

Second, four graduate students in psychology independently generated candidate items based on the qualitative data. Their contributions underwent iterative review and refinement, resulting in a preliminary pool of 40 items. All items were rated using a five-point Likert scale, with 1 indicating “not at all true” and 5 indicating “completely true” (see Appendix A).

Third, an expert evaluation procedure was implemented. Six specialists in special education and psychology assessed the relevance, clarity, and representativeness of each item. The Content Validity Index (CVI) was subsequently calculated, and items were revised based on expert feedback to strengthen content validity (see the revised version in Appendix B).

Fourth, a pilot study was carried out using Sample 2. The critical ratio (CR) was computed to evaluate item discrimination, and item–total correlations were examined to determine the degree to which each item was associated with the overall scale score. Cronbach’s *α* was calculated to assess the internal consistency reliability of the preliminary scale. Based on these analyses, item A7 was deleted, and the item pool was refined to a final set of 39 items.

#### 2.2.3 Exploratory factor analysis and confirmatory factor analysis

Exploratory factor analysis (EFA) was conducted using Sample 3 to examine the underlying structure of the scale. Item adjustments were made based on factor loadings and model fit. Subsequently, confirmatory factor analysis (CFA) was conducted using Sample 4 to validate the factor structure and further assess the psychometric properties of the finalized scale (see Table 2).

**Table 2:** Validity of expert content (*n* = 6).

Index	Content relevance	Content appropriateness	Clarity of expression	Content validity
SCVI	1	0.904545455	0.890909091	0.931818182
S-CVI/UA	1	0.975	0.9	0.9
S-CVI/Ave	1	0.904545455	0.890909091	0.901515152

## 3 Result

### 3.1 Validity of expert content

#### 3.1.1 Item analysis

Based on the results from Sample 2, the total scores of the 40 items across the five dimensions were ranked in descending order. The top 27% of participants were designated as the high-score group, while the bottom 27% constituted the low-score group. Since the data did not meet the assumption of normality, non-parametric tests (Mann–Whitney U test) were used to compare the mean scores of each item between the two groups. The results are presented in Appendix C.

Additionally, the correlation between each item score and the total scale score was calculated, as shown in Appendix C. Item A7 (*p* = 0.72) did not exhibit a significant difference between the high and low groups, indicating poor discriminative validity. Therefore, this item was excluded from subsequent analyses.

#### 3.1.2 Exploratory factor analysis

The principal component method was used to extract the factors, and the maximum variance orthogonal rotation was applied to determine the factor loading matrix. Items were deleted according to the following criteria:

1. Commonality below 0.30;
2. Factor loading below 0.40;
3. Multiple loadings, with all load values above 0.40 and load differences less than 0.30;
4. It was impossible to provide a reasonable explanation for the relationship between the items and the factors.

Based on these criteria, questions B4, D3, and E6 were deleted sequentially, leaving 36 items for analysis. See Table 3 for the results of the exploratory factor analysis (n = 195), and refer to Appendix D.

**Table 3:** Correlation between the initial item scores of the scale and the total score (*n* = 195).

Items	*r*	Items	*r*	Items	*r*
A1	0.557**	B7	0.583**	D4	0.711**
A2	0.572**	B8	0.595**	D5	0.625**
A3	0.571**	C1	0.628**	D6	0.644**
A4	0.549**	C2	0.579**	D7	0.629**
A5	0.530**	C3	0.614**	D8	0.571**
A6	0.500**	C4	0.608**	E1	0.603**
A8	0.561**	C5	0.680**	E2	0.521**
B1	0.674**	C6	0.570**	E3	0.542**
B2	0.624**	C7	0.591**	E4	0.587**
B3	0.506**	C8	0.566**	E5	0.604**
B4	0.474**	D1	0.668**	E6	0.472**
B5	0.645**	D2	0.584**	E7	0.589**
B6	0.645**	D3	0.485**	E8	0.522**

**p < 0.01.

#### 3.1.3 Confirmatory factor analysis

Confirmatory factor analysis was conducted on sample 4 using AMOS 21.0, and the results revealed that the fitting indicators of the model were good: *χ*^2^/df = 1.046, GFI = 0.910, RMR = 0.037, NFI = 0.909, NNFI = 0.995, IFI = 0.996, TLI = 0.995, PGFI = 0.798, PNFI = 0.843, PCFI = 0.923, SRMR = 0.037 CFI = 0.996, RMSEA = 0.012 (see Table 4).

**Table 4:** Results of confirmatory factor analysis (*n* = 338).

Index	x^2^	df	*p*	x^2^/df	GFI	RMSEA	RMR	CFI	NFI	NNFI
Standard	–	–	>0.05	<3	>0.9	<0.10	<0.05	>0.9	>0.9	>0.9
Value	610.803	584	0.214	1.046	0.91	0.012	0.037	0.996	0.909	0.995

Index	TLI	AGFI	IFI	PGFI	PNFI	PCFI	SRMR	RMSEA	90%	Cl
Standard	>0.9	>0.9	>0.9	>0.5	>0.5	>0.5	<0.1	–		
Value	0.995	0.897	0.996	0.798	0.843	0.923	0.037	0.011 ~ 0.022		

#### 3.1.4 Reliability analysis

Reliability analysis of the data results from Sample3 and sample 4revealed that the Cronbach’s Alpha coefficient of this scale ranged from 0.887 to 0.908, indicating good internal consistency. The correlation between all items and the total score was above 0.638. Deleting any one item did not significantly enhance the overall reliability, indicating that each question made a high contribution and could all be retained for subsequent analysis. Please refer to Table 5 for details.

**Table 5:** Results of reliability analysis.

Factor	Cronbach’s Alpha	Corrected Item-Total Correlation	Inter-Item Correlation
1. Neurophysiological drivers	0.887	0.65 ~ 0.69	0.47 ~ 0.57
2. Environmental and task adaptation	0.894	0.638–0.744	0.475–0.619
3. Motivation and rule cognition	0.908	0.674–0.731	0.512–0.593
4. Emotional and social interaction	0.905	0.685–0.752	0.535–0.633
5. Multi-support systems	0.902	0.679–0.740	0.513–0.625

#### 3.1.5 Validity analysis

##### 3.1.5.1 Structural validity

The results of confirmatory factor analysis initially indicated that the structure of this scale was reasonable. Correlation analysis of the data results from Sample 4 revealed that, in the attribution scale for behavioral problems of children with special needs, the correlation coefficients among various factors ranged from 0.36 to 0.52, which is within a reasonable range. The correlations between each factor and the total score of the scale were between 0.705 and 0.786, further indicating that the scale had good structural validity. Please refer to Table 6 for details.

**Table 6:** Results of internal correlation analysis of the attribution scale for behavioral problems of children with special needs (*n* = 195).

Factor	Factor 1	Factor 2	Factor 3	Factor 4	Factor 5
Factor 1	1	0.391***	0.360***	0.481***	0.443***
Factor 2	0.391***	1	0.498***	0.524***	0.455***
Factor 3	0.360***	0.498***	1	0.509***	0.427***
Factor 4	0.481***	0.524***	0.509***	1	0.420***
Factor 5	0.443***	0.455***	0.427***	0.420***	1
Total	0.705***	0.759***	0.762***	0.786***	0.725***

***p < 0.001.

## 4 Discussion

Grounded in ecological systems theory (Bronfenbrenner, 1979) and social cognitive theory (Bandura and Walters, 1977), this study systematically developed and rigorously validated the Attribution Scale for Behavioral Problems in Children with Special Educational Needs (CSEN) within the context of China’s advancing inclusive education policies and frontline practices. Unlike existing international assessment tools, the present scale integrates not only the structural and content strengths of mainstream global instruments but also critically incorporates context-specific attributional dimensions—such as policy environment and the multi-level coordination among families, schools, and the broader community—thereby enhancing both cultural relevance and practical applicability.

Through a staged, multi-level, and multi-sample empirical approach, the scale demonstrated excellent reliability and construct validity. Both exploratory and confirmatory factor analyses robustly supported the five-factor structure. The Cronbach’s alpha coefficients for the total scale and its subscales ranged from 0.88 to 0.91, and the inter-factor correlations (0.705–0.786, *p* < 0.001) confirmed strong internal consistency and discriminant validity. Model fit indices (CFI = 0.996, RMSEA = 0.012) further attest to the soundness of the scale’s factorial structure. Compared with internationally used measures, this scale offers stronger alignment with Chinese general and special education teachers’ practical attributional frameworks for behavioral problems in inclusive settings, underscoring its explanatory power and broad applicability.

Theoretically, this research marks the first local construction of a behavioral problem attribution model for CSEN within the framework of inclusive education in China, employing systematic qualitative and quantitative methodologies and multiple rounds of sample validation. This work substantially enriches the repertoire of assessment tools for Chinese special education and provides a standardized, empirically based instrument for behavioral identification, individualized intervention planning, and optimized resource allocation. Additionally, the scale establishes a unified comparative framework for cross-regional and cross-group studies.

In practice, the scale can be directly applied to school-based behavioral screening, evaluation of intervention strategies by class teachers and special educators, and evidence-based monitoring of inclusive education quality by educational administrators. Its use is expected to support professional development for educators and foster the advancement of high-quality inclusive education.

Nevertheless, the current sample was limited to Sichuan Province. Future research should seek to extend validation to diverse regions and school types nationwide to further assess and refine the generalizability of the instrument. It is also recommended to incorporate data from parents and students, enhancing the comprehensiveness and validity of attributions, and to adopt longitudinal designs for tracking test–retest reliability and intervention sensitivity.

Overall, the developed scale demonstrates both theoretical innovation and empirical rigor, representing a significant contribution to the scientific assessment of behavioral attributions in CSEN and providing robust support for the advancement of high-quality inclusive education in China.

## 5 Limitations

Despite the strengths of the present study, several limitations should be acknowledged. First, the sample was limited to teachers from Sichuan Province, which may limit the generalizability of the findings to other regions of China or internationally. While Sichuan represents a significant region in China, cultural, educational, and regional differences could influence the applicability of the scale in other contexts. Future studies should extend the validation process to diverse geographic regions and school types to assess the scale’s generalizability and cross-cultural applicability.

Second, although the study incorporated multiple samples, it relied heavily on teacher reports, which may be subject to response biases such as social desirability or self-report bias. Including data from additional sources, such as parents, students, and school administrators, could provide a more comprehensive understanding of the behavioral attributions and enhance the validity of the findings.

Third, the cross-sectional design of the study limits the ability to draw causal conclusions about the relationship between behavioral attributions and outcomes. Longitudinal studies are needed to track the stability of these attributions over time and assess their predictive power for long-term behavioral and academic outcomes in CSEN.

Lastly, while the scale demonstrated strong internal consistency and construct validity, further testing of its sensitivity to intervention and its potential to predict the effectiveness of various intervention strategies would strengthen its practical utility. Future research should explore the scale’s responsiveness to changes in behavioral interventions and its capacity to monitor the impact of different educational strategies on CSEN.

## Data Availability

The original contributions presented in the study are included in the article/Supplementary material, further inquiries can be directed to the corresponding authors.
