# Supplementary material for: Development of the attribution scale for behavioral problems in children with special educational needs: a reliability and validity study
Source: Front Psychol. 2026 Jan 13;16:1679823. doi: 10.3389/fpsyg.2025.1679823 (PMC12834769; doi:10.3389/fpsyg.2025.1679823)
Supplement: Supplementary file 1 [file Data_Sheet_1.pdf]

Appendix A

Student Behavioral Support Assessment Scale

Instructions:

Based on your daily observations of students' behavioral performance in the classroom, please choose the option that best matches the actual situation

(1 = Strongly Disagree, 5 = Strongly Agree).

All data will be used for academic research purposes only and will remain strictly confidential.

A. Neurophysiological Drivers

A1. The student generally has difficulty maintaining attention during class.

A2. The student frequently appears restless or unable to stay still.

A3. The student often seems tired or lacking in energy during lessons.

A4. The student shows poor physical coordination and struggles in activities requiring motor control.

A5. The student frequently displays involuntary small body movements when concentration is required.

A6. The student often experiences significant emotional fluctuations, such as anxiety or agitation.

A7. The student frequently exhibits excessive excitement or hyperactivity during activities.

A8. The student tends to appear lethargic or lacking vitality during class.

B. Task–Environment Adaptation

B1. The student's overall level of participation in classroom activities is relatively low.

B2. The student struggles to complete learning tasks within the allotted time.

B3. The student has difficulty consistently following classroom rules and behavioral expectations.

B4. The student easily becomes anxious or withdraws when facing challenging tasks.

B5. The student does not effectively utilize learning resources available in the classroom.

B6. The student adapts slowly to new learning situations or new tasks.

B7. The student often becomes confused or has difficulty transitioning between activities or tasks.

B8. The student tends to understand instructional content at a slower pace compared to peers.

C. Motivation, Rule Understanding, and Cognitive Regulation

C1. The student has insufficient understanding and mastery of classroom rules.

C2. The student finds it difficult to sustain interest and enthusiasm in classroom activities.

C3. The student frequently displays resistance or a negative attitude toward learning tasks.

C4. The student often procrastinates or avoids completing assigned classroom tasks.

C5. The student shows limited willingness to actively participate in learning activities.

C6. The student demonstrates weak curiosity or desire to explore new content.

C7. The student shows minimal response to incentives such as teacher praise or rewards.

C8. The student has noticeable difficulty accurately understanding teacher expectations and task requirements.

D. Emotional and Social Interaction

D1. The student seldom initiates interactions with peers during classroom activities.

D2. The student frequently engages in minor conflicts or disagreements with classmates.

D3. The student is reluctant to express personal thoughts or emotions.

D4. The student often appears socially isolated or passive.
D5. The student is not sensitive to others’ emotional needs or emotional changes.
D6. The student demonstrates low engagement in group work or cooperative activities.
D7. The student often appears tense, uneasy, or unnatural when interacting with peers.
D8. The student lacks effective skills or strategies for coping with social situations in the classroom.
E. Support System Development
E1. The student’s family provides limited support for learning and emotional development.
E2. The school offers insufficient individualized guidance and assistance inside and outside the curriculum.
E3. The student receives little positive feedback and counseling from teachers on campus.
E4. The student lacks constructive, mutually supportive relationships with peers.
E5. The student has difficulty expressing needs or seeking help when necessary.
E6. Community organizations or social resources play a minimal role in supporting the student’s learning.
E7. The school’s physical environment (classroom layout, learning spaces) provides inadequate support for student development.
E8. The campus cultural climate does not effectively promote the student’s sense of security, belonging, or confidence in personal growth.

Appendix B

Student Behavioral Support Assessment Scale (Teacher Version)

Instructions:

Based on your daily observations of students' behavioral performance in the classroom, please choose the option that best matches the actual situation

(1 = Strongly Disagree, 5 = Strongly Agree).

All data will be used for academic research purposes only and will remain strictly confidential.

A. Neurophysiological Drivers

A1. The student generally has difficulty maintaining attention during class.

A2. The student frequently demonstrates purposeless movement (such as pacing or repetitive bodily movements) in classroom settings.

A3. The student easily shows noticeably low energy or fatigue during classroom learning.

A4. The student shows insufficient physical coordination and has difficulty in tasks such as handwriting or physical activities.

A5. During classroom activities requiring focus, the student often unconsciously fiddles with stationery, clothing, or other objects.

A6. The student frequently experiences intense emotional changes without an obvious trigger and is easily anxious or agitated.

A7. During classroom interaction activities, the student often displays excessive excitement or difficulty calming down.

A8. The student generally appears lethargic and slow to respond during class.

B. Task–Environment Adaptation

B1. The student's overall level of participation in classroom activities is relatively low.

B2. The student has difficulty completing learning tasks within the allotted time.

B3. The student has difficulty consistently following classroom rules and behavioral expectations.

B4. When encountering challenging tasks, the student easily shows anxiety or actively gives up or avoids the task.

B5. The student cannot effectively utilize learning resources available in the classroom, such as textbooks, teaching aids, or multimedia materials.

B6. The student adapts slowly to new learning situations or new tasks.

B7. The student adapts slowly when transitioning between activities or tasks and efficiency is significantly reduced.

B8. The student understands instructional content at a slower pace than most other students in the class.

C. Motivation, Rule Understanding, and Cognitive Regulation

C1. The student has insufficient understanding and mastery of classroom rules.

C2. The student has difficulty maintaining sustained interest throughout the entire lesson.

C3. The student frequently displays resistance or a negative attitude toward learning tasks.

C4. The student often procrastinates in executing classroom tasks, or frequently requests to go to the restroom, as a means to avoid the task.

C5. The student demonstrates low enthusiasm for actively participating in classroom learning activities.

C6. The student has a low willingness to explore new knowledge or content, especially when task difficulty is high.

C7. The student shows minimal response to incentives such as teacher praise or rewards.
C8. The student has difficulty accurately understanding teacher expectations or task requirements and often requires repeated explanations to understand.
D. Emotional and Social Interaction
D1. In classroom social situations, the student seldom initiates interactions with peers.
D2. The student frequently experiences minor conflicts with peers, such as competing for objects or verbal disputes.
D3. The student rarely expresses or shares personal thoughts or feelings.
D4. The student often appears socially isolated or passive.
D5. The student finds it difficult to notice peers' emotional changes or does not pay sufficient attention to others' emotional needs.
D6. The student demonstrates low engagement in group work or cooperative activities.
D7. When interacting with peers, the student often shows signs of nervousness, discomfort, or unnatural emotional reactions.
D8. The student lacks effective skills or strategies for coping with social situations in the classroom, such as turn-taking or listening.
E. Support System Development
E1. The student's family provides limited time and frequency of involvement in supporting learning.
E2. The school provides insufficient individualized guidance inside and outside the curriculum and does not offer differentiated assignments or one-on-one tutoring tailored to specific student needs.
E3. The student receives little positive feedback and counseling from teachers on campus.
E4. The student lacks constructive, mutually supportive peer interactions in contexts such as group work or recess activities.
E5. The student has difficulty expressing needs or seeking help and shows weak awareness of actively asking for support.
E6. Community organizations or social resources (such as libraries or after-school activity centers) have low participation and limited roles in supporting the student's learning.
E7. School-provided basic support resources (such as classroom arrangement and learning spaces) are insufficient to meet diverse student needs and lack targeted adjustments.
E8. Teacher–student relationships and classroom cohesion do not effectively enhance students' sense of security, belonging, or confidence in personal growth.

Appendix C

Mann-Whitney U Test for score Differences of the initial items of the Scale between high and low groups (n=195)

Items	Mann-Whitney U	Asymp. Sig.(2-tailed)	Standard	Correlation Coefficient	Sig. (2-tailed)
A1	7	0.02	0.05	0.770	0.000
A2	5	<0.001	0.001	0.695	0.000
A3	8	0.002	0.01	0.659	0.000
A4	6	0.001	<0.01	0.678	0.000
A5	7	0.02	0.05	0.663	0.000
A6	1.5	<0.001	0.001	0.803	0.000
A7	21	0.72	>0.05	0.409	0.020
A8	6.5	0.02	0.05	0.717	0.000
B1	7	0.001	<0.01	0.679	0.000
B2	3	<0.001	<0.001	0.705	0.000
B3	3	<0.001	0.001	0.694	0.000
B4	0	<0.001	<0.001	0.788	0.000
B5	0	<0.001	<0.001	0.836	0.000
B6	2	<0.001	<0.001	0.652	0.000
B7	6.5	0.02	<0.05	0.583	0.000
B8	3	<0.001	<0.001	0.647	0.000
C1	10.5	0.05	⩽0.05	0.647	0.000
C2	1.5	<0.001	<0.001	0.808	0.000
C3	9	0.004	<0.01	0.730	0.000
C4	12	0.01	⩽0.01	0.629	0.000
C5	2	<0.001	<0.001	0.790	0.000
C6	3.5	<0.001	<0.001	0.766	0.000
C7	12	0.006	<0.01	0.555	0.001
C8	6	0.001	<0.01	0.676	0.000
D1	0	<0.001	<0.001	0.839	0.000
D2	15.5	0.018	<0.05	0.554	0.001
D3	0	<0.001	<0.001	0.724	0.000
D4	6	0.001	<0.01	0.775	0.000
D5	1.5	<0.001	<0.001	0.801	0.000
D6	6	0.001	<0.01	0.708	0.000
D7	10.5	0.006	<0.01	0.628	0.000
D8	3	<0.001	<0.001	0.682	0.000
E1	16	0.019	<0.05	0.506	0.003
E2	9.5	0.004	<0.01	0.553	0.001
E3	14.5	0.015	<0.05	0.500	0.004
E4	0	<0.001	<0.001	0.731	0.000
E5	15.5	0.022	<0.05	0.364	0.041

E6	5	0.001	<0.01	0.601	0.000
E7	15.5	0.014	<0.05	0.419	0.017
E8	18.5	0.032	0.05	0.470	0.007

Appendix D

Results of Exploratory Factor Analysis (n=195)

items	factor1	factor2	factor3	factor4	factor5	Communality
A1				0.753		0.640
A2				0.706		0.613
A3				0.708		0.605
A4				0.744		0.622
A5				0.716		0.602
A6				0.738		0.597
A8				0.751		0.651
B1		0.678				0.648
B2		0.677				0.602
B3		0.762				0.632
B5		0.670				0.614
B6		0.755				0.697
B7		0.790				0.702
B8		0.805				0.723
C1	0.686					0.598
C2	0.749					0.624
C3	0.764					0.667
C4	0.738					0.628
C5	0.724					0.675
C6	0.757					0.632
C7	0.779					0.686
C8	0.724					0.591
D1			0.721			0.667
D2			0.705			0.610
D4			0.742			0.744
D5			0.788			0.715
D6			0.675			0.605
D7			0.715			0.630
D8			0.715			0.596
E1					0.760	0.674
E2					0.654	0.505
E3					0.738	0.621
E4					0.723	0.627
E5					0.716	0.634
E7					0.716	0.612
E8					0.788	0.680
Eigenvalue	5.463	4.645	4.617	4.577	4.520	
% of Variance	14.007	11.911	11.838	11.737	11.589	
Cumulative %	14.007	25.918	37.757	49.494	61.083	
